# Discovery of New Catalytic Topoisomerase II Inhibitors for Anticancer Therapeutics

**DOI:** 10.3389/fonc.2020.633142

**Published:** 2021-02-01

**Authors:** Victor M. Matias-Barrios, Mariia Radaeva, Yi Song, Zaccary Alperstein, Ahn R. Lee, Veronika Schmitt, Joseph Lee, Fuqiang Ban, Ning Xie, Jianfei Qi, Nada Lallous, Martin E. Gleave, Artem Cherkasov, Xuesen Dong

**Affiliations:** ^1^ The Vancouver Prostate Centre, Department of Urologic Sciences, University of British Columbia, Vancouver, BC, Canada; ^2^ Department of Geriatrics, Union Hospital, Tongji Medical College, Huazhong University of Science and Technology, Wuhan, China; ^3^ Department of Biochemistry and Molecular Biology, University of Maryland, Baltimore, Baltimore, MD, United States

**Keywords:** topoisomerase II, catalytic inhibitor, androgen receptor, prostate cancer, computer aided drug design

## Abstract

Poison inhibitors of DNA topoisomerase II (TOP2) are clinically used drugs that cause cancer cell death by inducing DNA damage, which mechanism of action is also associated with serious side effects such as secondary malignancy and cardiotoxicity. In contrast, TOP2 catalytic inhibitors induce limited DNA damage, have low cytotoxicity, and are effective in suppressing cancer cell proliferation. They have been sought after to be prospective anticancer therapies. Herein the discovery of new TOP2 catalytic inhibitors is described. A new druggable pocket of TOP2 protein at its DNA binding domain was used as a docking site to virtually screen ~6 million molecules from the ZINC15 library. The lead compound, T60, was characterized to be a catalytic TOP2 inhibitor that binds TOP2 protein and disrupts TOP2 from interacting with DNA, resulting in no DNA cleavage. It has low cytotoxicity, but strongly inhibits cancer cell proliferation and xenograft growth. T60 also inhibits androgen receptor activity and prostate cancer cell growth. These results indicate that T60 is a promising candidate compound that can be further developed into new anticancer drugs.

## Introduction

Drugs targeting DNA topoisomerase II (TOP2) are widely used in clinics for a variety of hematological and solid tumors (1, 2). TOP2 catalyzes sequential steps of enzymatic reactions that cleave and re-ligate double-strand DNA (dsDNA) breaks to relieve the superhelical state of genomic DNA or disentangle interlinked chromosomes (3, 4). Human cells express TOP2A and TOP2B genes, among which TOP2A is essential for cancer cell division because it is expressed at the S and M phases of the cell cycle (5, 6), and is required for DNA replication and chromosome segregation for mitosis (7, 8). TOP2B is dispensable for cell viability (9, 10) and its expression is independent of the cell cycling (5, 6). However, it is often hijacked by oncogenic transcriptional factors (11–13) that confer cancer cells a proliferative transcriptome. Inhibitors that block both TOP2 isoforms could serve as a double-bolt lock to effectively inhibit cancer cell growth.

TOP2 inhibitors are classified into two types: TOP2 poisons and catalytic inhibitors (1, 4, 14). Poisons (*e.g.*, etoposide) stabilize the covalent TOP2–DNA cleavage complex, resulting in DNA damage that can cause cell death (4, 15). Unfortunately, the mechanism of action (MOA) of TOP2 poisons makes them often associated with serious side effects such as secondary malignancy and cardiotoxicity (16, 17). It had been shown that etoposide treatment to patients can induce acute myeloid leukemia (t-AML) (16), which is attributed to TOP2B-, but not TOP2A-mediated DNA damage that disrupts the mixed lineage leukemia (MLL) gene and MLL associated genes in benign bone marrow cells (16). Since TOP2B is expressed and remains active during the G1 phase of the cell cycle in non-proliferating benign cells, TOP2 poisons cannot differentiate cancer cells from benign cells when introducing genotoxic effects.

In contrast, catalytic inhibitors do not induce the covalent TOP2–DNA cleavage complex and cause minimal DNA damage, but inhibit TOP2 activity through various mechanisms of actions (MOAs) such as blocking TOP2 binding to DNA (18); inhibiting ATP binding to TOP2 (19), or stabilizing the non-covalent TOP2–DNA complex (20). These inhibitors have low genotoxicity but can still effectively suppress cell proliferation. These findings rationalize that designing catalytic TOP2 inhibitors may be more selective to inhibit tumor cell growth and cause lower genotoxicity to non-dividing benign cells in the human body. Catalytic inhibitors are sought after as prospective anticancer drugs to be used alone or in combination with existing chemotherapies (21).

However, currently available catalytic inhibitors have serious off-target effects, poor stability, and low potency (22–24). None of them has yet advanced into clinical practice. Almost all TOP2 inhibitors including these used in clinics are not developed by structure-based rational drug design, but by high throughput screening of toxic scaffolds (15, 25–27). Many TOP2 inhibitors are DNA intercalators (*e.g.*, mitoxantrone and doxorubicin) that indirectly block TOP2 enzyme activity. The resultant genotoxic and cytotoxic effects are inevitably not fully related to TOP2 inhibition. Some catalytic inhibitors (*e.g.*, merbarone and BNS-22) are TOP2 binders but have no accurate information of ligand–protein interactions, making it challenging to perform further medicinal chemistry (22–24, 28, 29). Merbarone is proven to be a catalytic inhibitor that prevents the formation of the TOP2–DNA cleavage complex (23). However, it also confusingly induces DNA breaks and causes S and G2/M arrest with undefined MOA to date (30) and had failed several clinical trials (24, 31). We hypothesize that structure-based rational drug design technology can lead to discoveries of new catalytic inhibitors that have more defined MOA and are more feasible to perform medicinal chemistry to improve their potency and pharmacological properties (32). This technology leverages rapid advancement in protein crystallography and computational chemistry to screen candidate compounds *in silico* based on the crystal structure of the target proteins (33–36).

In this study, we have launched a computer-aided drug discovery (CADD) campaign to screen ~6 million molecules from the ZINC15 database (37) against a newly characterized docking pocket on the surface of TOP2 proteins. It was facilitated by the implementation of consensus scoring from various virtual screening programs (33–36). We found that the lead compound T60 and its derivatives are catalytic inhibitors. They have low cytotoxicity, but strongly suppress cancer cell proliferation, suggesting that T60 is a promising candidate to be developed into anticancer drugs.

## Materials and Methods

### *In Silico* Virtual Screening for Potential TOP2 Binders

#### Protein Preparation

The 4FM9 PDB entry for TOP2A with 2.901 Å resolution (38) was downloaded from PDB (https://www.rcsb.org). It was pre-processed using the Protein Preparation Wizard build in Maestro Schrödinger software (39). The H-bond assignments were optimized using PROPKA with pH value of 7.0 (40). The protein was minimized using OPLS3 force field parameters with a default constraint of 0.30 Å RMSD for heavy atoms (41). To predict potential docking sites, the MOE Site Finder package was used to search the protein surface with virtual atom probes, and the Pock Drug software was used to calculate the pocket volume (42).

#### *In Silico* Screening

ZINC15 is a database containing >800 million molecules, among which ~6 million drug-like molecules (37) were downloaded with the additional criteria that they must be purchasable. A Schrödinger’s Grid generation program (34) was used to calculate a docking grid. The Glide software was used to perform the docking with default parameters (36). The choice of docking software was governed by a previous assessment of the docking programs where Glide was one of the best software (43), and our previous studies in which we had successfully applied the Glide and eHiTS software to identify an AR inhibitor (44). The docking site residues were used from the binding site selected by the site finder algorithm from MOE. The top 100,000 molecules that were scored and ranked by GlideSP (36) were subjected to re-docking using the eHiTS program (34). To perform consensus scoring [as it was shown to improve hit rates and binding pose prediction accuracy (45)], we calculated RMSD between docking poses predicted by Glide and eHiTS and selected molecules with RMDS below 2 Å. To exclude molecules with potential toxicity, the ADMET software was used to calculate the ADMET_Risk, Tox_Risk, and CYP_Risk descriptors, where molecules were selected if they had scored less than 6.5, 3.3, and 1 respectively as suggested by the software (46). After the lead compound was validated by a series of bioassays, the ROCS shape similarity program offered by Open Eye was used to search similar molecules with the Implicit Dean Mills force field was used with all other parameters as default (47).

#### Molecular Dynamics

To evaluate the stability of the docking pocket and the molecular origins of binding, we performed molecular dynamics (MD) simulation by using the Desmond simulation package of Schrödinger (48). An SPC water solvent model and orthorhombic simulation box shape were applied. The NPT ensemble was used with a pressure of 1.01325 bar and temperature 300 K. The simulations lasted for 30 ns. The protein–ligand system reached equilibration (**Figure S1B**). The protein RMSD was measured with superimposition on protein C-alpha atoms, while the ligand RMSD was measured on ligand heavy atoms. The first frame was used as a reference.

### Chemicals

ZINC compounds were purchased from Enamine, Life Chemicals Ltd, Princeton Biomolecular Research, and Vitas. T60 and T60 derivatives were synthesized by Life Chemicals Ltd. with purity >95%.

### *In Vitro* TOP2 Activity Assays

#### K-DNA Decatenation Assays

The human TOP2 assay kit (#TG1001, TopoGEN, Buena Vista, CO) and TOP2B (HTB205, Inspiralis, UK) were used for K-DNA decatenation assays. TOP2 enzymes were incubated with 250 ng of kDNA in a reaction buffer containing 50 mM Tris-HCl (pH 8.0), 150 mM NaCl, 10 mM MgCl2, 2 mM ATP, 0.5 mM DTT, and 30 mg/ml BSA in the presence of candidate compounds or etoposide and ICRF193 as control inhibitors in a final volume of 20 µl. The reaction was incubated at 37C for 2 h after which 4 µl stop solution (5× buffer is 5% Sarkosyl, 0.125% bromophenol blue, 25% glycerol) was added. The reaction was resolved by electrophoresis on a 0.8% agarose gel at 60 V and stained with 0.5 µg/ml ethidium bromide for 15 min. DNA bands were visualized using a gel imager (Gel Doc™ EZ System) and quantified using the Image Studio™ Lite Quantification Software.

#### DNA Relaxation Assays

The human TOP2A and TOP2B were used to perform DNA relaxation assays. TOP2 enzyme was incubated with the supercoiled pHOT-1 plasmid in a reaction buffer containing 50 mM Tris-HCl (pH 8.0), 150 mM NaCl, 10 mM MgCl2, 2 mM ATP, 0.5 mM DTT, and 30 mg/ml BSA in the presence of candidate compounds or etoposide and ICRF193 as control inhibitors in a final volume of 20 µl. The reaction was incubated at 37C for 2 h and stopped by adding 2 µl of 10% SDS and 1 µl of 250 mM of EDTA, followed by the addition of 50 µg/ml of proteinase K at 45 C for 1 h. The reaction was then resolved by electrophoresis on a 1% agarose gel at 80 V and stained with 0.5 µg/ml ethidium bromide for 15 min. DNA bands were visualized using a gel imager (Gel Doc™ EZ System) and quantified using the Image Studio™ Lite Quantification Software.

### Ethidium Bromide Displacement Assays

Ethidium bromide displacement assay was performed as previously described (49, 50) using Amsacrine (mAMSA) as a positive control. In a 20 µl reaction, compounds were mixed with 100 µg/ml of salmon sperm DNA and 2 µg/ml of ethidium bromide in a fluorescence buffer containing 10 mM HEPES, pH 7.9, 100 mM KCl, 5 mM MgCl2, 0.1 mM EDTA, pH 8.0. Triplicate reactions were subjected to fluorescence emission of 590 nm and excitation of 535 nm. Signals were detected by using the Multimode Microplate Reader, Infinite^®^ F500.

### DNA Cleavage Assays

TOP2A or TOP2B was incubated with the supercoiled pHOT-1 plasmid in a reaction buffer containing 50 mM Tris-HCl (pH 8.0), 150 mM NaCl, 10 mM MgCl2, 0.5 mM DTT, and 30 mg/ml BSA in the presence of candidate compounds or etoposide and ICRF193 as control inhibitors in a final volume of 20 µl. The reactions were incubated in 37C for 30 min and stopped by adding 2 µl of 10% SDS and 1 µl of 250 mM of EDTA, followed by the addition of 50 µg/ml of proteinase K at 45C for 30 min. The reaction was then resolved by electrophoresis on a 1% agarose gel containing 0.5mg/ml ethidium bromide in TAE buffer at 60 V. DNA bands were visualized using a gel imager (Gel Doc™ EZ System) and quantified by using the Image Studio™ Lite Quantification Software.

### Bio-Layer Interferometry Assays

BLI assays were performed as we previously reported (33). The direct and reversible interactions between small molecules and TOP2 protein were quantified by BLI using OctetRED (ForteBio, Bohemia, NY). Human TOP2A (431–1,193) was produced *in situ* with AviTag technology. The AviTag sequence (GLNDIFEAQKIEWHE) followed by a six-residue glycine serine linker (GSGSGS) was incorporated with TOP2A (431–1193). *Escherichia coli* BL21 containing both biotin ligase and TOP2A (431–1193) vectors were induced with 0.5 mM isopropyl-*β*-D-thiogalactopyranoside (IPTG) and biotin at 16C overnight. The bacteria were then lysed by sonication, and the resulting lysate was purified by immobilized metal ion affinity chromatography (IMAC) with nickel nitrilotriacetic acid (NiNTA) resin and cation-exchange chromatography (HiTrap SP). Purified TOP2A (431–1193) (50 μg/ml) was bound to the super-streptavidin sensors over 50 min at room temperature. The sensor was kept in an assay buffer [20 mM N-2-hydroxyethylpiperazine-N0 -2-ethanesulfonic acid (HEPES), 150 mM NaCl, 500 μM tris(2-carboxyethyl) phosphine (TCEP), and 1% dimethylsulfoxide (DMSO)], when small molecules were added. After 200 s, a wash buffer was used to flush the sensor plate for 100 s. BLI signal was recorded throughout the 300 s following the manufacture’s instruction from OctetRED (ForteBio, Bohemia, NY).

### Cell Lines and Cell Culture

LNCaP, PC3, DU145, K562, Jurkat, THP, HeLa, H-446, NCI-H82, NT2, NCCIT, U937, HEK293T, and NCI-H69 cell lines were purchased from the American Type Culture Collection (ATCC; Manassas, VA, USA). MR49F cell line was provided by Dr. Martin Gleave (Vancouver Prostate Centre, Canada). BPH-1 cell line was provided by Dr. Simon Hayward (Vanderbilt University, TN, USA). LNCaP95 was a generous gift from Dr. Alan Meeker (John Hopkins University, MD, USA). NT2 and NCCIT cells were generous gifts from Dr. Leendert Looijenga (3rd) (University Medical Center Rotterdam). Jurkat, H-69, K562, H-446, THP, U937, H-82, NCCIT, LNCaP, BPH-1, and MR49F were cultured in RPMI-1640 with 10% Fetal Bovine Serum (FBS). Additionally, MR49F cells were maintained in media with 10 μM enzalutamide. LNCaP95 cells were cultured in phenol-free RPMI-1640 medium with 5% charcoal stripped serum (Hyclone). HeLa, NT2, 293T, PC3, and DU145 were cultured in DMEM supplemented with 10% FBS. All cell lines used in this study tested negative for mycoplasma contamination and were authenticated by short tandem repeat assays.

### Cell Proliferation, Cytotoxicity, FACS, and Immunoblotting Assays

Cell proliferation rates were measured by using the CellTitre 96 AqueousOne kit (Promega) and bromodeoxyuridine (BrdU) assay kit (Millipore) according to the manufacturer’s protocol as we have described (51). Cytotoxicity was evaluated by using the commercial kit (CAT# 8078, ThermoFisher). Culture media was collected and used to measure lactate dehydrogenase (LDH) levels by a colorimetric method following the manufacture’s protocol (ThermoFisher). Cell cycling was assessed by using BrdU incorporation into S-phase DNA by using the APC BrdU flow kit (BD Pharmingen; Franklin Lakes, New Jersey) according to the manufacturer’s protocol. 1 mM of BrdU was added to cells and incubated for 6 h. Cells were incubated with anti-BrdU antibody and stained with 7-AAD before processing the cells for flow cytometry (52). Immunoblotting assays followed the standard protocol as we have reported (51–53).

### Thermal Shift, Chromatin Fractionation, and Immunoblotting Assays

In the thermal shift assays, cells were treated with vehicle or T60 for 1 h and split into equal aliquots to be heated at 37 to 46°. Protein lyses were collected to perform immunoblotting assays with TOP2 antibodies (Abcam 12318, Santa Cruz sc-13059 & sc-365071). Our chromatin fractionation assays followed the protocol as previously described (Méndez & Stillman, 2000; Wysocka, Reilly, & Herr, 2001). Briefly, K562 cells (4 × 10 (7) cells/ml) were collected and re-suspended in buffer A (10 mM HEPES [pH 7.9], 10 mM KCl, 1.5 mM MgCl2, 0.34 M sucrose, 10% glycerol, 1 mM DTT, and protease inhibitor cocktail), before Triton X-100 was added to final concentration at 0.1%. Cells were then incubated on ice for 10 min, and nuclei were collected by centrifugation at speed of 1,300g for 4 min at 4°C. The nuclei were then washed once with buffer A and then incubated with buffer B (3 mM EDTA, 0.2 mM EGTA, 1 mM DTT, and protease inhibitor cocktail [ROCHE]) for 30 min in ice. Insoluble chromatin was collected by centrifugation at 1,700g for 4 min at 4°C, and washed once with buffer B. Chromatin associated proteins were extracted by incubation with Buffer C [500 mM Tris-HCl (pH 6.8), 500 mM NaCl, and protease inhibitor cocktail (ROCHE)] followed by brief sonication. The supernatant was collected to perform immunoblotting assays with TOP2 antibodies as we have described (51–53).

### Electrophoresis Mobility Shift Assay

EMSA followed the standard protocols as we and others described with minor modifications (54–56). The double-strand DNA oligo, 5ZRF (AGC CGA GCT GCA GCT CGG CT labeled by IRDye700) was purchased from IDT (Coralville, USA). Human TOP2 (431–1193), TOP2A (TopoGEN) or TOP2B (Inspiralis) proteins were incubated with 300 nM of 5ZRF oligo in a standard 20 µl EMSA reaction in a reaction buffer containing 20 mM HEPES-NaOH, pH 7.4, 2 mM DTT, 3 mM MgCl2, 0.05% NP-40, protease inhibitors, and 0.1 µg poly[dI:dC]. After incubation for 30 min at 37C, reactions were loaded onto 4% non-denatured polyacrylamide gel in 0.5× TBE buffer. The DNA oligo bands were visualized by using the Li-Cor Odyssey 9120 Infrared Imaging System.

### Confocal Microscopy

Cells were fixed with 4% paraformaldehyde, treated in 0.25% Triton X-100 for 15 min, incubated with F-actin conjugated to Phalloidin-iFluor 488 (Abcam; UK), and mounted with DAPI staining mount (Vector Labs; Burlingame, USA). Cell images were captured by confocal microscopy at 63× magnification (Zeiss; Germany) (51, 53).

### Animal Studies

The polymeric paste was prepared with 50% poly-DL-lactide-coglycolide (PLGA) and 50% PEG 300 (w/w) as we have reported (57). Compounds (*e.g.* etoposide and T60) were incorporated as powders using a mortar and pestle to reach the targeted concentrations. The density of the paste was determined to be 1.2 g/ml ( ± 3%). One million K562 cells were inoculated subcutaneously in bilateral flanks of nude mice (Nu/Nu). Once tumors reached 100–200 mm^3^, mice were randomly selected for a single-dose treatment with 30 µl paste containing 0.3% etoposide, 1% T60 or T633, or an equivalent volume of vehicle. Tumor volumes were measured every three days and were calculated by length × width × depth × 0.5236. Mice were euthanized when tumor volumes reached 15% of body weight or weight loss >20% occurred. The remaining mice were euthanized after the control group was euthanized. All animal procedures were under the guidelines of the Canadian Council on Animal Care and were approved by the UBC Animal Care Committee (# A19-0256).

### Statistics

Statistical analysis was performed using the GraphPad Prism 5.01 software (GraphPad Software, CA, USA). Differences between the two groups were compared by student t-test. One-way ANOVA followed t-test was used to compare differences among multiple groups. The levels of significance were set at p < 0.05 as *, p < 0.01 as **, and p < 0.001 as ***.

## Results

### Identification of a Novel Docking Pocket for TOP2 Binders

**Figure 1:**
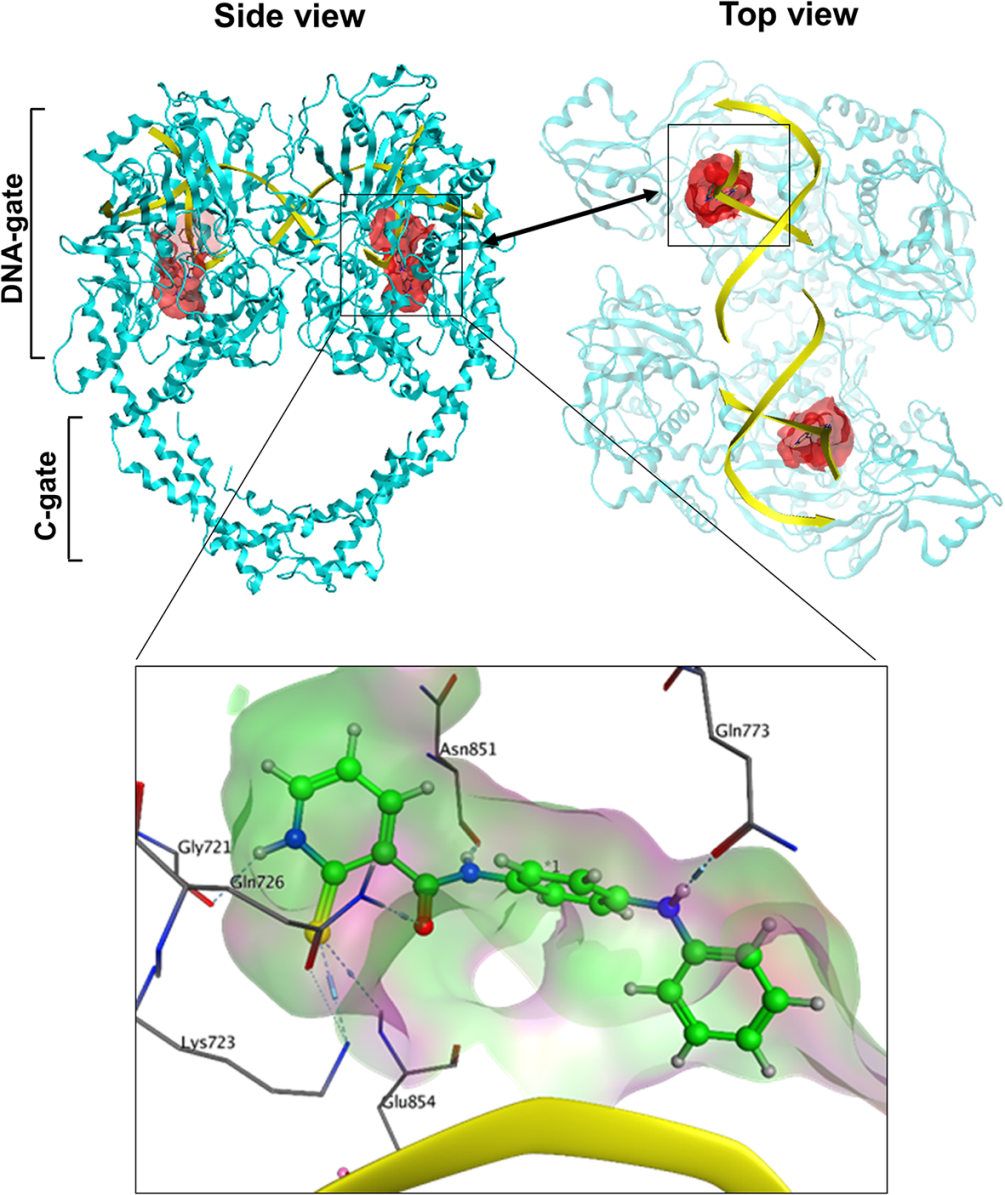
The spatial relationship of the docking pocket with TOP2 and DNA. Both top and side views show the relative position of TOP2A dimer (cyan), DNA substrate (yellow), and the molecular surface of the docking pocket (red) used for in silico drug screening.

**Figure 2:**
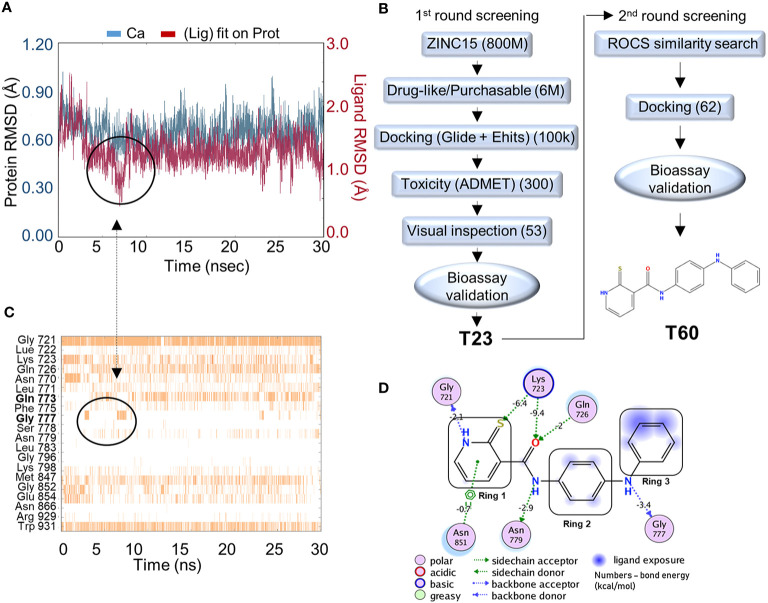
*In silico* screening for TOP2 inhibitors. **(A)** Molecular dynamics simulation of the docking pocket (blue) and T60 inside the TOP2A docking pocket (red) in 30ns. **(B)** A computer-aided drug design (CADD)_pipeline was applied to screen for TOP2 binders. Two rounds of virtual screening protocol combined with K-DNA decatenation and relaxation bioassays led to the discovery of the lead compound, T60. **(C)** A bar chart of protein–ligand contacts during the 30 ns simulation period. The interchanging bond formation (circled) between Gln773 and Gly777 is corresponding to T60 RMSD (circled) from **(A)** in the first 8 ns. **(D)** A schematic diagram shows T60 atoms interacting with surrounding protein residues of TOP2A. Bond energies were shown in kcal/mol.

Since we hypothesized that chemicals that interfere with TOP2 from binding their DNA substrates would likely be catalytic inhibitors, we focused on the potential docking sites at the TOP2–DNA interface. We analyze the TOP2A–DNA structure 4FM9 (38) by using the MOEs Site-Finder software as we previously reported (58–60), and found a deep binding pocket at the TOP2A–DNA binding interface (**Figure 1**). The pocket is surrounded by polar residues (Gly721, Lys723, Gln726, Gln773, Asn779, Asn851, Gly852, and Arg929), and non-polar residues (Leu722, Lue771, Ala852, and Trp931) from TOP2A. It is highly homologous between TOP2A and TOP2B with only one amino acid different from each other (Leu722/Phe738, TOP2A/TOP2B). Since both amino acids are non-polar, we believe this pocket would serve as a drug-binding site for both TOP2A and TOP2B. Its convex hull volume is 1671.88 Å that may accommodate a wide range of ligands with potential for ligand modification and optimization. We have assessed the stability of the docking pocket by a molecular dynamics’ simulation through 30 ns molecular dynamics and found that the pocket (blue line) changed minimally (<1 Å) (**Figure 2A**). These results indicate that this docking pocket is stable and favorable as a druggable site.

### *In Silico* Screening and Biological Validation of Candidate Compounds

To screen for TOP2 binders, we initiated a CADD campaign. We first filtered the ZINC15 library down to ~6 million chemicals by two criteria: 1) are purchasable, and 2) have drug-like properties determined by Lipinski’s rule of 5 (61) (**Figure 2B**). These molecules were docked into the identified TOP2A pocket with GlideSP (36). The top 1.5% of compounds (~100,000 compounds) were selected, which cut-off was primarily dictated by the capacity of downstream filtering. These compounds were re-docked with another docking software, eHiTS (34). We then compared the docking poses predicted by both software to select compounds with consensus docking poses. This combination of docking software was proven effective and fast by our previous studies (58, 59). Molecules that were flagged as being potentially toxic or having poor pharmacokinetics by ADMET software (62, 63) were excluded. We then visually inspected the dataset to discard molecules with incorrectly built structures, improper protonation states, or unrealistic binding poses. At the end of this first round of screening, 53 candidate molecules were selected to be tested using *in vitro* TOP2A kinetoplast DNA (K-DNA) decatenation and supercoiled plasmid relaxation assays. We found that the compound T23 to be the most potent chemical to inhibit TOP2 activities.

A new round of *in silico* screening was then initiated using T23 as a stating scaffold for compounds with better activity profiles (**Figure 2B**). We screened the ZINC15 library for T23 analogs using a shape similarity search program ROC (47). Molecules with high similarity scores were docked into the pocket using GlideSP to discard those with implausible docking poses (36, 37). At the end of the second round of screening, 62 molecules were selected for *in vitro* TOP2 K-DNA decatenation and plasmid relaxation assays that led to the discovery of the most active lead compound, designated as T60.

### Molecular Dynamics of T60 Within the Docking Pocket

To assess the stability of T60–TOP2A interaction *in silico*, a molecular dynamics simulation was used (64). T60 was observed to sit stably within the pocket throughout the simulation (**Figure 2A**). The RMSD of the ligand (red line) heavy atoms showed some fluctuations at the first 8 ns followed by relatively fixed positioning within ~1 Å. This initial fluctuation was caused by interchangeable H-bond formation of Ring 3 with Gln773 and Gly777 as shown on the protein–ligand contact timeline (**Figure 2C**). The Ligand Root Mean Square Fluctuation (L-RMSF) was also calculated to assess ligand per atom fluctuations through the simulation (**Figure S1**). Ring 1 atoms were relatively fixed mainly due to a persistent H-bond with Gly721, which was sustained throughout 89% of the simulation (**Figure 2C**). All hydrogen donors and acceptors of T60 make H-bond contacts with the surrounding residues, suggesting that T60 has a high affinity to the pocket (**Figure 2D**). These results indicate that T60 is stably positioned in the docking pocket where Rings 1 and 2 fit deep inside the pocket and Ring 3 is extended out facing DNA.

### T60 Is a Catalytic TOP2 Inhibitor

**Figure 3:**
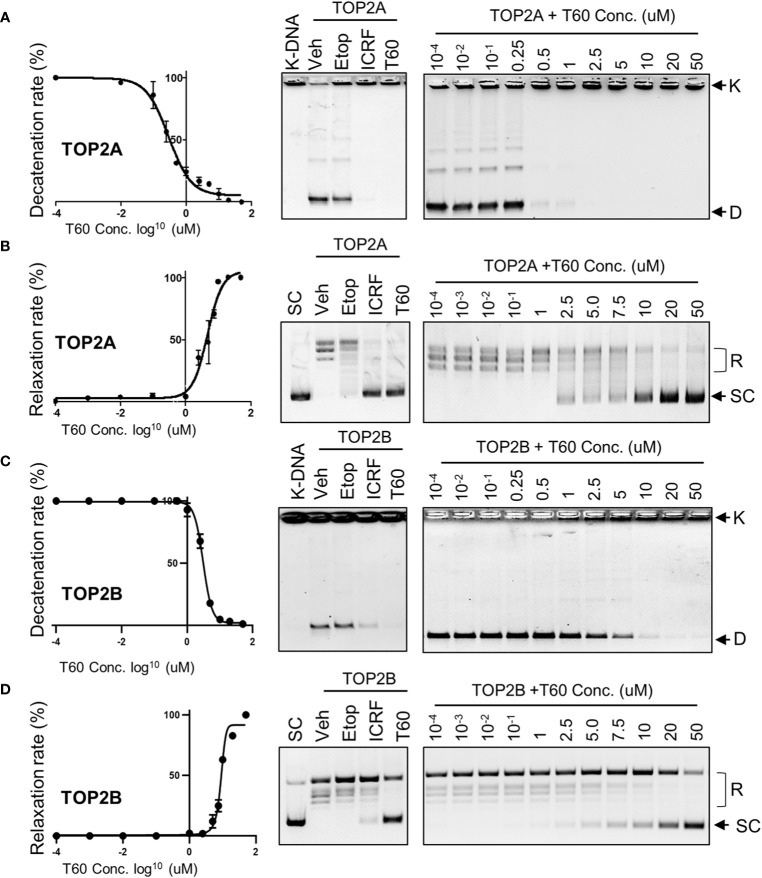
T60 is an inhibitor of TOP2A and TOP2B. K-DNA decatenation assays measured TOP2A **(A)** and TOP2B **(C)** activities that were inhibited by T60. The decatenation assays were repeated three times per concentration, and the densitometry of the D-DNA bands was used to establish an inhibition curve for IC_50_ calculation. Etoposide and ICRF193 were used as positive controls. Plasmid DNA relaxation assay measured TOP2A **(B)** and TOP2B **(D)** activities that were inhibited by T60. The relaxation assays were repeated three times per concentration and the densitometry of the SC bands was used to establish an inhibition curve for IC_50_ calculation. K, catenated kinetoplast DNA; D, decatenated K-DNA; SC, supercoiled forms of the plasmid; and R, relaxed forms of the plasmid.

**Figure 4:**
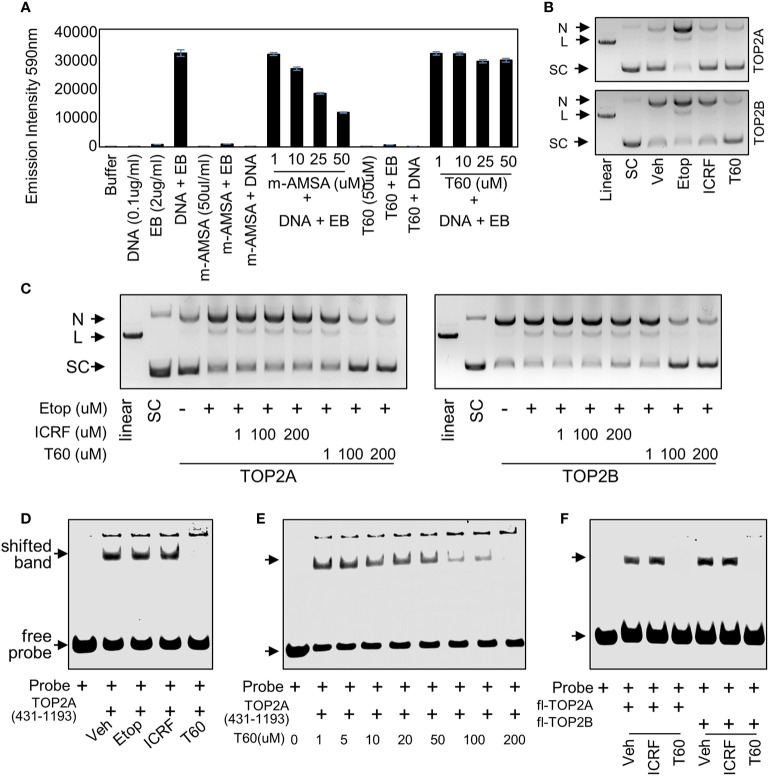
T60 is a catalytic TOP2 inhibitor **(A)** In ethidium bromide displacement assays, 100 µg/ml salmon sperm DNA was mixed with or without 2 µg/ml ethidium bromide together with the indicated chemicals. Reactions were subjected to fluorescence emission of 590 nm and excitation of 535 nm. Obtained signals were detected by using the Multimode Microplate Reader (Infinite F500). **(B)** DNA cleavage assays were performed using human TOP2A or TOP2B incubated with the supercoiled pHOT plasmid in the presence of the vehicle, 200 µM of etoposide, ICRF193, or T60 in a reaction buffer without ATP. **(C)** DNA cleavage assays were performed using full-length human TOP2A and TOP2B incubated with the supercoiled pHOT plasmid in the presence of 200 µM of etoposide plus 1, 100, and 200 µM of ICRF193 or T60. Reactions from **(B, C)** were separated on a DNA gel with ethidium bromide. **(D, E)** EMSA was performed by incubating 30 µM of the purified human TOP2A(431-1193) with 300nM of IRDay700 labeled 5ZRF DNA oligo. Vehicle or 200 µM of etoposide, ICRF193, or T60 was added as shown in **(D)**, or 0**–**200 µM of T60 was added in the reactions as shown in **(E)**. **(F)** EMSA was performed by incubating 2 µM of full-length TOP2A or TOP2B with 300 nM of IRDay700 labeled 5ZRF DNA oligo in the presence of vehicle or 200 µM of ICRF193 or T60. All experiments were repeated three times, and one representative image was shown. N, nicked DNA; L, linear DNA; and SC, supercoiled DNA.

To validate that T60 is a TOP2 inhibitor, we used K-DNA decatenation assays and showed that ICRF193 and T60, but not etoposide, inhibited TOP2A mediated K-DNA decatenation (**Figure 3A**). K-DNA (K) consists of thousands of interlocked circular DNA molecules, which can be decatenated by TOP2 enzymes. The amount of decatenated circular DNA (D) reflects TOP2 activity. We showed that T60 inhibits TOP2A activity in a dose-dependent manner with an IC_50_ at ~0.3 µM (**Figure 3A**). DNA relaxation assays showed that ICRF193 and T60 inhibited TOP2A activity in changing the supercoiled (SC) into the relaxed (R) forms of plasmid DNA (**Figure 3B**). T60 inhibited this TOP2A activity with IC_50_ at ~4.7 µM. Since the docking pocket is almost identical between TOP2A and TOP2B, we observed similar effects of T60 in inhibiting TOP2B activity with IC_50_ at ~3.0 µM and ~8.9 µM in the decatenation and relaxation assays, respectively (**Figures 3C, D**). TOP2A and TOP2B are purchased from two different sources. Since we did observe that TOP2B is more active than TOP2A even though the same units were used, we are reluctant to conclude that T60 is more effective to suppress TOP2A than TOP2B. To exclude the possibility that T60 interacts with DNA by forming intercalation complexes thereby inhibits TOP2 activities, we performed ethidium bromide displacement assays (**Figure 4A**). The fluorescence induced by the ethidium bromide/DNA complex was reduced by increasing doses of m-AMSA (a reference intercalator), but not T60. These results showed that T60 does not intercalate with DNA and supported that T60 binds TOP2 to inhibit TOP2 activities. Together, these assays validated our *in-silico* prediction by the CADD pipeline that T60 is a TOP2 inhibitor.

To further test whether T60 is a catalytic TOP2 inhibitor, we performed DNA cleavage assays in which SC plasmid was incubated with etoposide, ICRF193, or T60 in the presence of TOP2A or TOP2B in a reaction buffer with no ATP (**Figure 4B**). The reactions were separated by DNA agarose gels with ethidium bromide. We found etoposide but not ICRF193 and T60 induced detectable linear plasmid on the gel, indicating that T60 does not act as etoposide that stabilizes the covalent TOP2–DNA cleavage complex. To determine whether T60 prevents the TOP2–DNA cleavage formation or acts similarly to ICRF193 that stabilizes the non-covalent TOP2–DNA complex after etoposide induces the TOP2–DNA cleavage complex, we performed DNA cleavage assays in the presence of etoposide. We found that co-treatment of T60 but not ICRF193 prevented etoposide-induced DNA cleavage in reactions containing either TOP2A or TOP2B (**Figure 4C**). These results confirmed that T60 exerts its inhibitory effects by blocking TOP2 from establishing the covalent TOP2–DNA cleavage complex.

To further characterize how T60 blocks the formation of the covalent TOP2–DNA cleavage complex, we performed electrophoresis mobility shift assay (EMSA) to test whether T60 blocks TOP2 from binding to DNA, because the T60 docking pocket is at the TOP2–DNA interaction interface. Human TOP2A protein (aa 431–1193) was expressed from BL21plyss bacteria (**Figure S2A**) and purified, using which to incubate with an IR700-labeled dsDNA oligo containing a consensus TOP2 binding site. TOP2A (431–1193) induced an upshifted band, which can be abolished by T60, but not etoposide and ICRF193 (**Figure 4D**). This T60 action is in a dose-dependent manner (**Figure 4E**). Furthermore, we replaced TOP2A (431–1193) with full-length human TOP2A and TOP2B protein (TopoGEN and Inspiralis respectively) in the EMSA. We observed consistent results that both TOP2 proteins formed mobility retarded bands that can be interrupted by T60 but not ICRF193 (**Figure 4F**). These results indicate that T60 prevents TOP2 from interacting with DNA. In summary, these results indicated that T60 acts as a TOP2 catalytic inhibitor, and its MOA is to disrupt the TOP2–DNA interaction thereby prevent the TOP2–DNA cleavage formation.

### Structure–Activity Relationship Between T60 and TOP2

**Figure 5:**
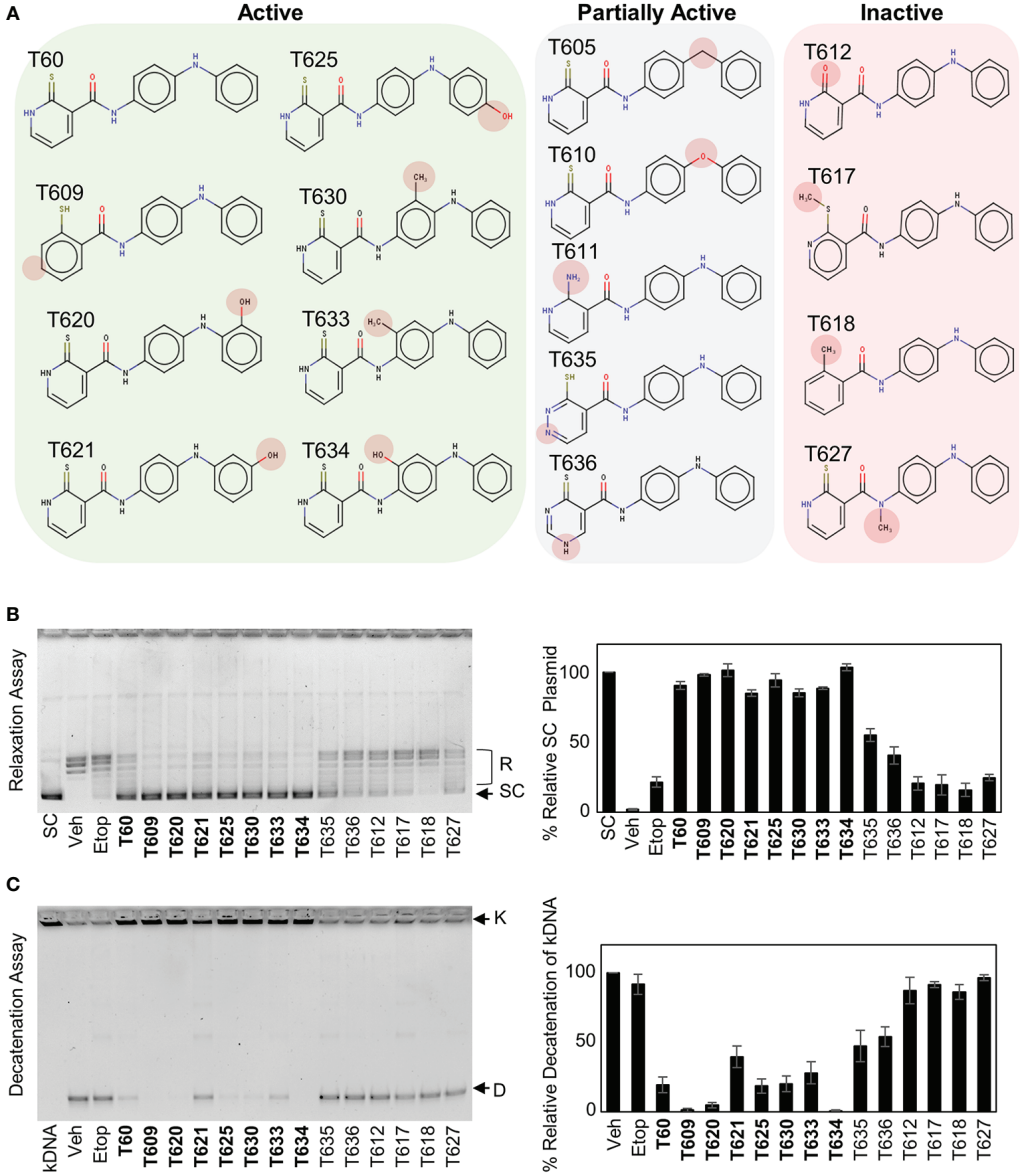
Structure–activity relationship (SAR) between T60 and TOP2. **(A)** T60 derivatives were custom synthesized (>95% purity) and tested for their effects on TOP2 by both relaxation and K-DNA decatenation assays. **(B, C)** Representative results of relaxation **(B)** and K-DNA decatenation **(C)** assays were presented. The densitometry of SC and D-DNA bands were used to establish the efficacy of the suppressive effects of T60 and its derivatives to TOP2A. Experiments were repeated three times, and results were presented as mean ± SD.

Understanding the key structural elements of ligand–target interaction is fundamental for the optimization of lead compounds. We have used T60 as a backbone to design a series of derivatives to explore the corresponding SAR (**Figure 5**). Analyzing T60 derivatives had led to several critical observations *in silico*. The unsaturated sulfur attached to Ring 1 forms one strong hydrogen bond with Lys723 that is important for T60 activity since its replacements render the compounds (T611, T612, T617, and T618) inactive. The ligand potency decreases with the increased polarity of Ring 1, which is observed in compounds in the following order: T609, T60, T635, and T636. The most active compound T609 lacks N atom on Ring 1; T60 with one N atom is less active, while T635 and T636 with two N atoms are nearly inactive. The two nitrogen atoms located on the linkers between the rings form crucial hydrogen bonds with the protein residues. Replacement of –NH groups with –NMe in T627 led to a complete abolishment of T60 activity. T630, T633, and T634 showed stronger activities than T60, which could be attributed to the presence of –OH and –Me fragments that could be accommodated by an adjacent protein cavity (**Figure 2**). Notably, T634 is most active among the three due to the formation of the hydrogen bond between its hydroxyl group and Asn851 backbone. Similarly, T620 and T625 carrying a hydroxyl group on the right ring exhibited increased activity due to the formation of an additional H-bond as indicated (**Figure S3**). These *in silico* observations warrant further investigations using structural biology techniques such as X-ray crystallography.

To test whether T60 and its derivatives bind TOP2 in a reversible and dose-dependent manner, we performed Bio-Layer Interferometry (BLI) assays. TOP2A (431–1193) was purified (**Figure S2A**), biotinylated, and immobilized onto streptavidin biosensors. Sensors were then dipped in successive wells containing increasing concentrations of T633. The binding of T633 to TOP2A (431–1193) changed the spectral pattern of reflected light. BLI curves indicated a reversible and dose-response binding of T633 to TOP2 (**Figure S2B**) affirming a non-covalent binding as predicted by our *in-silico* simulation studies (**Figure 2**).

### T60 Inhibits Cancer Cell Proliferation and Tumor Xenograft Growth

**Figure 6:**
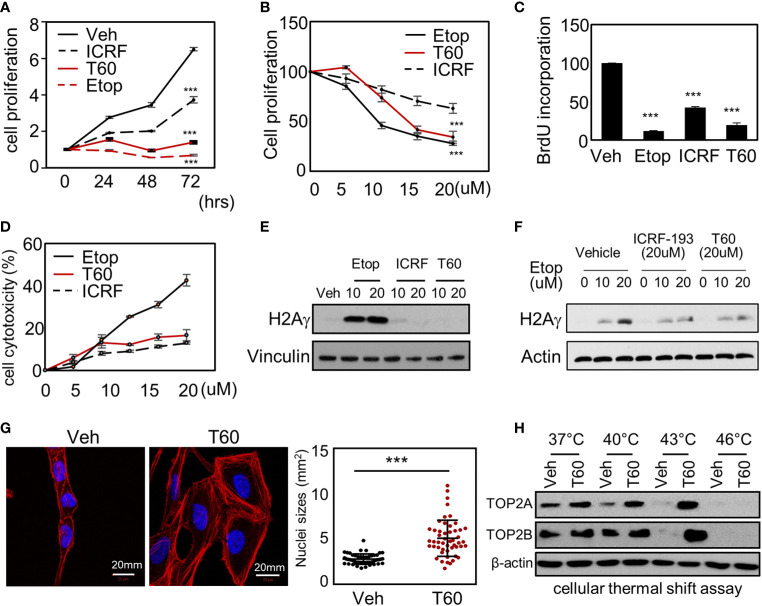
T60 targets TOP2 in cells and suppress cell proliferation. **(A)** K562 cells were treated with 20 µM of etoposide, ICRF193, or T60 for 0, 24, 48, and 72 h. **(B)** K562 cells were treated with 0, 5, 10, 20 µM of etoposide, ICRF193, or T60 for 48 h. Cell proliferation rates were measured by MTS assays. **(C)** HeLa cells were treated with the vehicle or 20 µM of T60, etoposide, or ICRF193 for 48 h. BrdU incorporation rates were measured. **(D)** HeLa cells were treated with 0**–**20 µM of etoposide, ICRF193, or T60 for 48 h. Cytotoxicity was evaluated by measuring the LDH levels from the culture media. **(E)** K562 cells were treated with the vehicle or 10**–**20 µM of etoposide, ICRF193, or T60 for 4 h. Immunoblotting measured p-H2AX*γ* levels in cells with vinculin as a loading control. **(F)** K562 cells were treated with the vehicle or 10**–**20 µM of etoposide and co-treated with vehicle, 20 µM ICRF193, or 20 µM T60 for 4 h. Immunoblotting measured p-H2AX*γ* levels in cells with beta-actin as the loading control. **(G)** HeLa cells were treated with either vehicle or 20 µM T60 for 48 h, before co-stained with DAPI (blue) and phalloidin (red) and examined by confocal microscopy. Fifty cells from five random high power fields were used to analyze nuclei sizes by the Image J software. All results were repeated in three independent experiments. **(H)** K562 cells were treated with the vehicle or 20 µM T60 for 2 h and used to perform cellular thermal shift assays between 37 and 46°. Protein levels of TOP2A and TOP2B were measured by immunoblotting with beta-actin as the loading control. All experiments were repeated three times, and results were presented as mean ± SD. One-way ANOVA followed by *t*-test was used to determine differences between groups with the level of significance set at *p < 0.05 and **p < 0.01. One-way ANOVA followed by t-test was used to determine differences between groups with the level of significance set at p < 0.001 as ***.

To study the effects of T60 on cell proliferation, we showed that T60 inhibited K562 cell proliferation in a dose- and time-dependent manner (**Figures 6A, B**). Its suppressive effects were stronger than ICRF193 and were comparable to etoposide. Because TOP2 is required for DNA replication during the S phase of cell cycling, we performed BrdU incorporation assays to show that T60 reduced DNA replication in HeLa cells (**Figure 6C**). FACS assays further confirmed that T60 treatment resulted in the cell population reduced at the G1 phase, but accumulated at S and G2/M phases of Hela cells (**Figure S4**). However, T60 exerts low cytotoxicity in great contrast to etoposide shown by LDH cytotoxicity assays, which measured the release of the lactate dehydrogenase into culture media by dead cells after exposure to cytotoxic reagents (**Figure 6D**). T60 did not cause intracellular DNA damage reflected by phospho-H2AX*γ* protein levels (**Figure 6E**), but inhibited etoposide-induced phospho-H2AX*γ* protein levels (**Figure 6F**). These results were consistent with our findings that T60 prevents the formation of TOP2–DNA cleavage complex (**Figures 4B–D**). Confocal fluorescence microscopy results showed that T60 treated Hela cells had enlarged cell bodies and nuclei (**Figure 6G**) that could be explained by which T60 interferes TOP2A from segregating chromosomes during mitosis. To confirm T60 acts on TOP2 proteins in the cells, we performed thermal shift assays. TOP2A and TOP2B proteins were stabilized up to 43C in Hela cells treated with 20 µM of T60, but only to 40C in the vehicle treated cells (**Figure 6H**). The chromatin fragmentation assays further confirmed that TOP2A and TOP2B association with chromatin was reduced by T60, while the total TOP2 protein levels remained unchanged (**Figure S5**). These results together indicate that T60 has low toxicity but strongly inhibits cancer cell proliferation by targeting TOP2 proteins.

**Figure 7:**
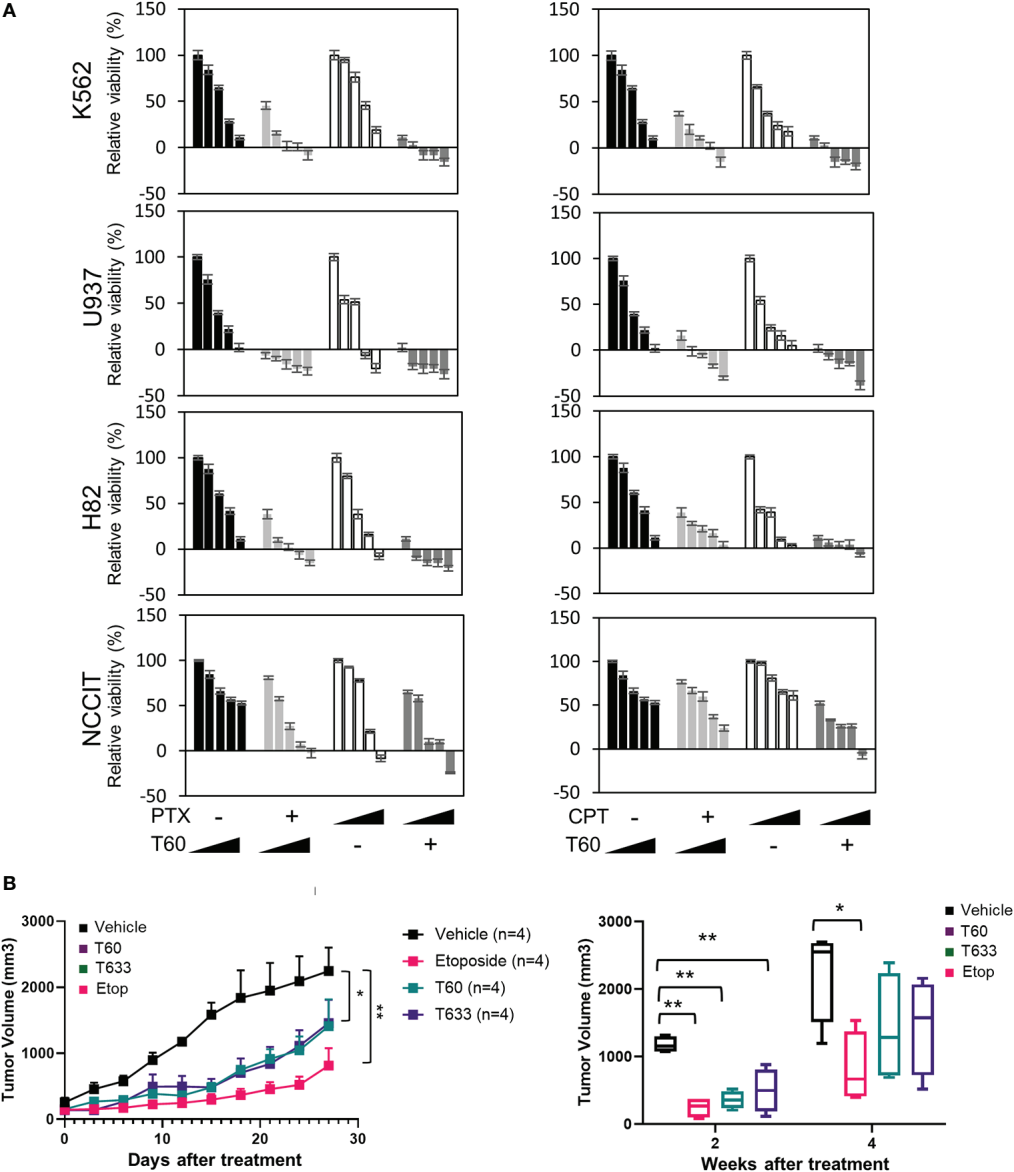
T60 inhibits cell growth of multiple cancer cell types. **(A)** K562, U937, H82, and NCCIT cancer cells were treated with increasing doses of paclitaxel or camptothecin alone or in combination with 0, 5, 10, 15, and 20 µM of T60. The concentration of paclitaxel and camptothecin were indicated in **Supplementary Table 1**. Cell viability was determined by MTS assays. Results were calibrated with vehicle treatment as 100%. **(B)** K562 xenografts were injected with polymeric paste containing vehicle, 1% T60 or T633, or 0.3% etoposide (n = 4/group). Tumor volumes were measured and plotted. Results are presented as the mean ± SEM. One-way ANOVA followed by *t*-test was used to compare between groups with the level of significance set at *p < 0.05 and **p < 0.01. ns: no significance.

We have also tested T60 inhibitory effects in multiple cancer cell models from leukemia, small cell lung cancer, and testicular cancer *etc* (**Figures 7A** and **S6**). T60 treatment alone exerted suppressive effects on all cell models, though the extent of suppression varied among cell models. T60 completely inhibited cell growth of U937 leukemia cells, but only 50% NCCIT testicular cells. These results may be possibly explained in part by the variable doubling time of each line under *in vitro* culture conditions. Importantly, the suppressive effects of T60 in all cell models were not accompanied by DNA damage shown by phospho-H2AX*γ* expression (**Figure S7**). Since both paclitaxel and camptothecin target cancer cell division, we showed that T60 enhanced the effectiveness of these drugs in suppressing cancer cell growth in all tested cancer cell lines (**Table S1**). T60 can lower the doses of paclitaxel and camptothecin to achieve maximum inhibition to cancer cells, suggesting that co-treatment of T60 may enhance the effectiveness of these chemotherapy drugs, but lower genotoxicity for patients.

To further assess T60 suppressive effects to tumors, we used a polymeric paste mixed with T60 (or its derivative T633) to enable sustained local releases of the drugs *via* intra-tumor injection of K562 xenografts. We previously reported that this paste can release incorporated drugs in a sustained and steady manner for about two weeks (57), while the paste itself has no systemic or local toxic effects. This method helps provide proof-of-principle of tumor inhibitory effects of drug candidates directly and excludes possible metabolic or tissue distributional factors that may complicate experiment result interpretation. T60, T633, and etoposide inhibit tumor growth over the four-week time course (**Figure 7B**). Both T60 and T633 strongly inhibit tumor growth comparable to etoposide at the end of two weeks. At the end of four weeks, tumor volumes in the etoposide, but not the T60 and T633 groups were statistically lower than the vehicle group possibly reflecting that a complete consumption of T60 and T633 after the two-week treatment. These findings indicate that sustained exposure of T60 and T633 to tumor cells can inhibit tumor growth *in vivo*.

### T60 Inhibits AR Signaling and PCa Cell Growth

**Figure 8:**
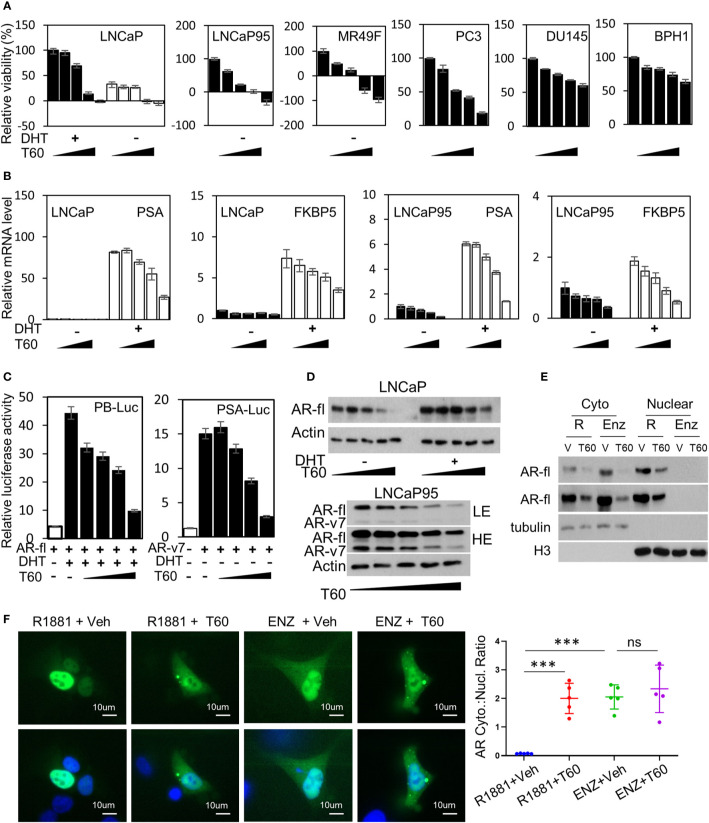
T60 inhibits AR signaling and PCa cell growth. **(A)** PCa and BPH1 cell lines were treated with 0, 5, 10, 15, and 20 µM of T60. LNCaP cells were also co-treated with −/+10nM DHT. Cell proliferation rates were measured by MTS assays. Results were calibrated with vehicle treatment as 100%. **(B)** LNCaP and LNCaP95 cells were treated with −/+10nM DHT and 0, 5, 10, 15, 20 µM of T60. PSA and FKBP5 mRNA levels were measured by real-time PCR with GAPDH as the housekeeping gene. **(C)** 293T cells were transfected with probasin- or PSA-luciferase reporter plasmid together with either AR full length or AR-v7 expression plasmid. Cells were treated with vehicle, 10 nM DHT, or 0, 5, 10, 15, and 20 µM of T60. Luciferase activity was measured and calibrated by renillar luciferase activity. **(D)** LNCaP and LNCaP95 cells were treated with −/+10 nM DHT and 0, 5, 10, 15, and 20 µM of T60. **(E)** LNCaP cells were treated with either 1nM R1881 or 5 µM enzalutamide in the presence of −/+ 20 µM T60 for 24 h. Cytoplasmic and nuclear proteins were extracted and used to perform western blotting with AR antibody with tubulin and Histone H3 as the loading controls. **(F)** DU145 cells were transfected with the EGFP-AR plasmid. Cells were treated with either 1nM R1881 or 5 µM enzalutamide in the presence of −/+ 20 µM T60 for 24 h. Subcellular localization of AR protein was observed by microscopy. In each treatment group, five cells from random fields were used to measure fluorescence intensity by the Image J software. Cytoplasmic:nuclear ratio = (total fluorescence - nuclear fluorescence)/nuclear fluorescence. Results are presented as the mean ± SEM. One-way ANOVA followed by *t*-test was used to compare among groups with the level of significance set at ***p < 0.001.

To test the effects of T60 on PCa cells, we have used five PCa and the BPH1 benign cell lines. The LNCaP cells are reliant on the androgen/AR signaling. The LNCaP95 line is derived from LNCaP cells and becomes androgen-independent but AR-dependent. The MR49F model is also derived from LNCaP cells but has developed resistance to enzalutamide *via* an F877L mutation in its ligand-binding domain (65). The AR in the MR49F cells carries therapy-resistant mutations that enable AR to be transcriptionally active. We observed that T60 strongly suppressed LNCaP, LNCaP95, and MR49F cell viability, and even caused cell death at high concentrations (**Figure 8A**). T60 also suppressed PC3, DU145, and BPH1 cell viability but to a much less extent compared to AR-positive PCa cell lines. These results indicate that AR-positive cells are more sensitive to T60 inhibition, likely because T60 exerts dual suppressive effects on both PCa cell proliferation and AR functions. T60 inhibited mRNA expression of AR target genes such as PSA and FKBP5 in a dose-dependent manner (**Figure 8B**). T60 inhibited luciferase activities driven by AR controlled probasin and PSA promoters in 293T cells transfected with AR or AR-v7 expression vector (**Figure 8C**). It inhibited the protein levels of AR full-length (AR-fl) and AR-v7 (**Figure 8D**). T60 reduced nuclear localization of AR proteins in LNCaP cells (**Figure 8E**) and GFP-AR transfected DU145 cells (**Figure 8F**) similar to that under enzalutamide treatment. These results together suggest that the AR cannot be stably recruited to its targeted promoters because T60-mediated TOP2B inhibition prevents the relaxation of superhelical genomic DNA. Consequently, AR proteins are exported to the cytoplasm for protein degradation. Together, these results indicated that T60 inhibits both PCa cell growth and AR activity.

## Discussion

This study reports an application of rational drug design to discover T60 as a new catalytic TOP2 inhibitor. Our *in house* CADD pipeline had demonstrated its capability to screen large databases with accurate docking software that allows the identification of candidate compounds such as T60 in a time- and cost-effective manner. Inhibitors with a novel MOA can be identified using these computational tools. As a result, T60 is found to be the first-in-kind inhibitor that is not a DNA intercalator but blocks TOP2 binding to DNA. T60 inhibits cancer cell proliferation with minimal genotoxicity. It has dual suppressive effects on both AR activity and AR-positive PCa cell growth, highlighting that it is a promising candidate for further drug development.

TOP2 poisons rely on creating DNA damage to suppress tumor growth. The drawback of this MOA is that TOP2 induced DNA damage also occurs in non-cancerous cells. Even though the non-homologous end joining (NHEJ) pathway may repair the DNA damage and rescue the cells, it leaves genomic mutations, deletions, and gene rearrangements that are often oncogenic. Examples are etoposide and teniposide, which cause mutations of the MLL gene on chromosome 11q23 and therapy-related secondary leukemia (66). Furthermore, if the tumor cells survived TOP2 poison treatment, they become even more difficult to be treated due to their heterogeneous genomic alterations. In contrast, T60 uses alternative MOA to block TOP2 activity and cell proliferation but causes minimal DNA breaks and low cytotoxicity. More importantly, it can lower the doses of other chemotherapy drugs to achieve maximal cell growth inhibition. These results support that T60 and its derivatives may provide clinical benefit when used alone or in combination with other anticancer drugs.

The suppressive effects of T60 to AR may also have clinical benefit in treating PCa. While all AR inhibitors approve by the FDA target the ligand-binding domain (LBD) of AR to prevent AR from activation, tumor cells develop therapy-resistance by either overexpressing AR or synthesizing AR splice variants or drug-resistant AR mutants to resume AR signaling. Because TOP2B is essential for all AR isoforms to initiate transcription even after they are activated and recruited to targeted promoters under antiandrogen treatment conditions, blocking TOP2B serves as a new approach to inhibit both AR and AR-driven PCa growth. We showed that AR-positive PCa cell models are particularly sensitive to T60 treatment. T60 inhibits AR activity through multiple mechanisms including i) T60 mediated TOP2B inhibition prevents AR from initiating gene transcription (**Figure 8C**); ii) T60 inhibits AR protein expressions (**Figure 8D**), and iii) T60 promotes cytoplasm localization of AR proteins (**Figures 8E, F**). The latter two aspects are likely due to T60-mediated TOP2B inhibition that prevents nuclear AR to be stably recruited on the target promoters, resulting in AR protein translocation to the cytoplasm for protein degradation. Together these results support that T60 represents a new class of AR inhibitors through a mechanism different from all currently known anti-androgens.

Our studies had also revealed several novel aspects of the structural biology of TOP2. We reported a new docking pocket at the interface between TOP2 and DNA that can be used to perform CADD to discover new TOP2 inhibitors. Although TOP2 undergoes sequential conformation changes when processing DNA substrates, our molecular dynamics simulation experiments showed that this docking pocket has high stability (**Figure 2**), suggesting it could be accessible to ligands despite dynamic conformations of the TOP2 protein. However, when T60 sits inside this pocket, it can block TOP2 from interacting with DNA (**Figure 4**). Since DNA binding is the initial step for TOP2 to catalyze superhelical DNA, T60 and its derivatives can also interfere with subsequent ATP hydrolysis when TOP2 catalyzes its DNA substrates as reported (67). These findings provide new perspectives of TOP2 molecular biology through understanding the interactions between ligands and the newly discovered docking pocket.

In summary, we identify T60 as a promising drug candidate that suppresses cancer cell proliferation with low cytotoxicity. T60 inhibits AR activity and AR-driven PCa cell growth. These findings warrant further investigation for T60 to be developed into anticancer therapy.

## Data Availability Statement

The raw data supporting the conclusions of this article will be made available by the authors, without undue reservation.

## Ethics Statement

The animal study was reviewed and approved by UBC Animal Care Committee, University of British Columbia, Vancouver, BC, Canada (# A19-0256).

## Author Contributions

Conceptualization: XD and AC. Investigation and Discussion: VM-B, MR, YS, ZA, AL, NX, and JQ. Writing: XD, AC, and MG. Funding Acquisition: XD, and AC. Resources: VS, JL, FB, and NL. Supervision: XD, AC, and MG. All authors contributed to the article and approved the submitted version.

## Conflict of Interest

The authors declare that the research was conducted in the absence of any commercial or financial relationships that could be construed as a potential conflict of interest.
