# Supplementary material for: Discovery of New Catalytic Topoisomerase II Inhibitors for Anticancer Therapeutics
Source: Front Oncol. 2021 Feb 1;10:633142. doi: 10.3389/fonc.2020.633142 (PMC7883873; doi:10.3389/fonc.2020.633142)
Supplement: Supplementary file 1 [file Table_1.docx]

**Supplementary Figures and Tables**

**
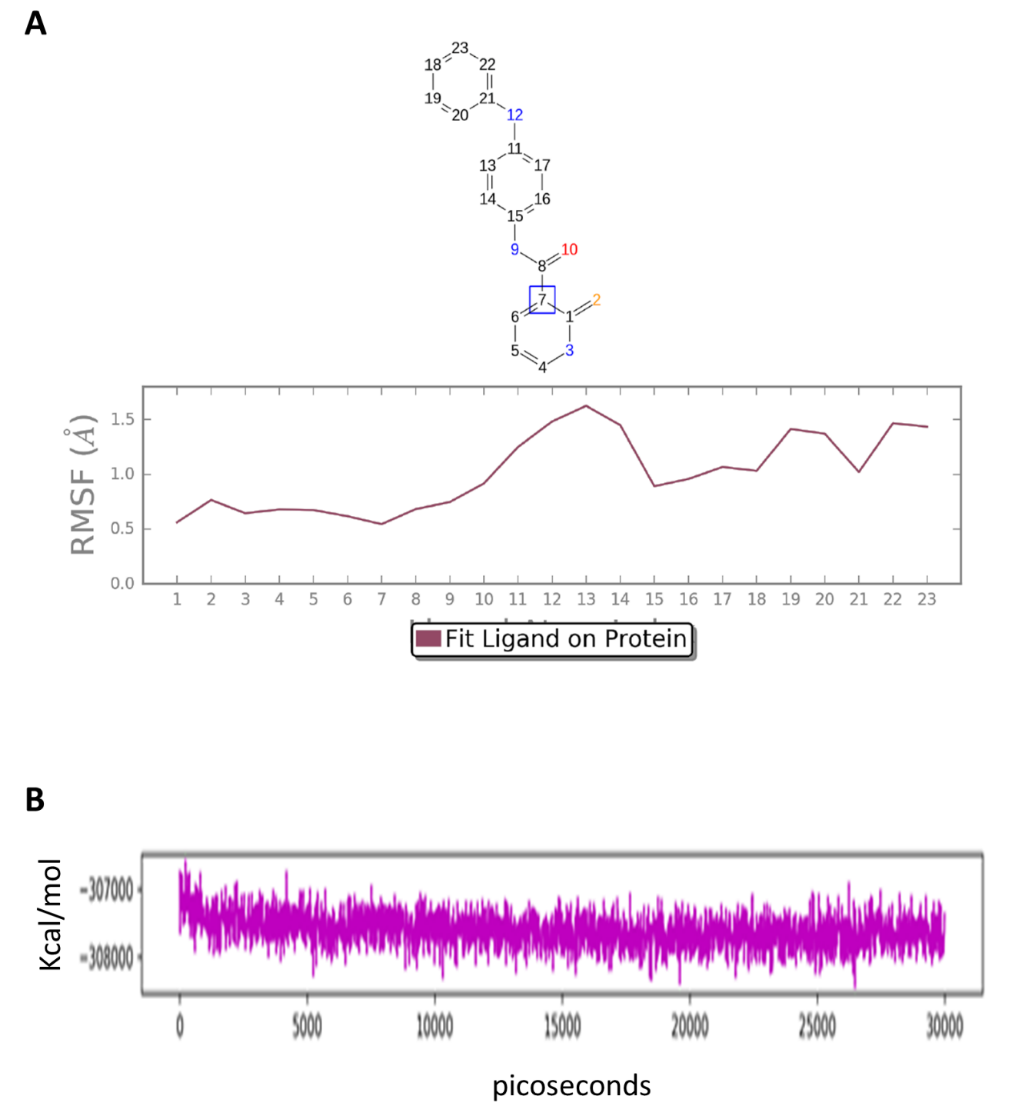
**

**Supplementary Figure 1. Molecular dynamics of T60 in the docking pocket. (A)** The Ligand Root Mean Square Fluctuation (L-RMSF) of T60 during the 30 ns molecular dynamics simulation. Note the relatively fixed positioning of atoms 1-10 indicating strong H-bonding. (**B)** Total energy (kcal/mol) of the protein-ligand system throughout the 30 ns simulation (x-axis in picoseconds). The equilibration was reached.

**
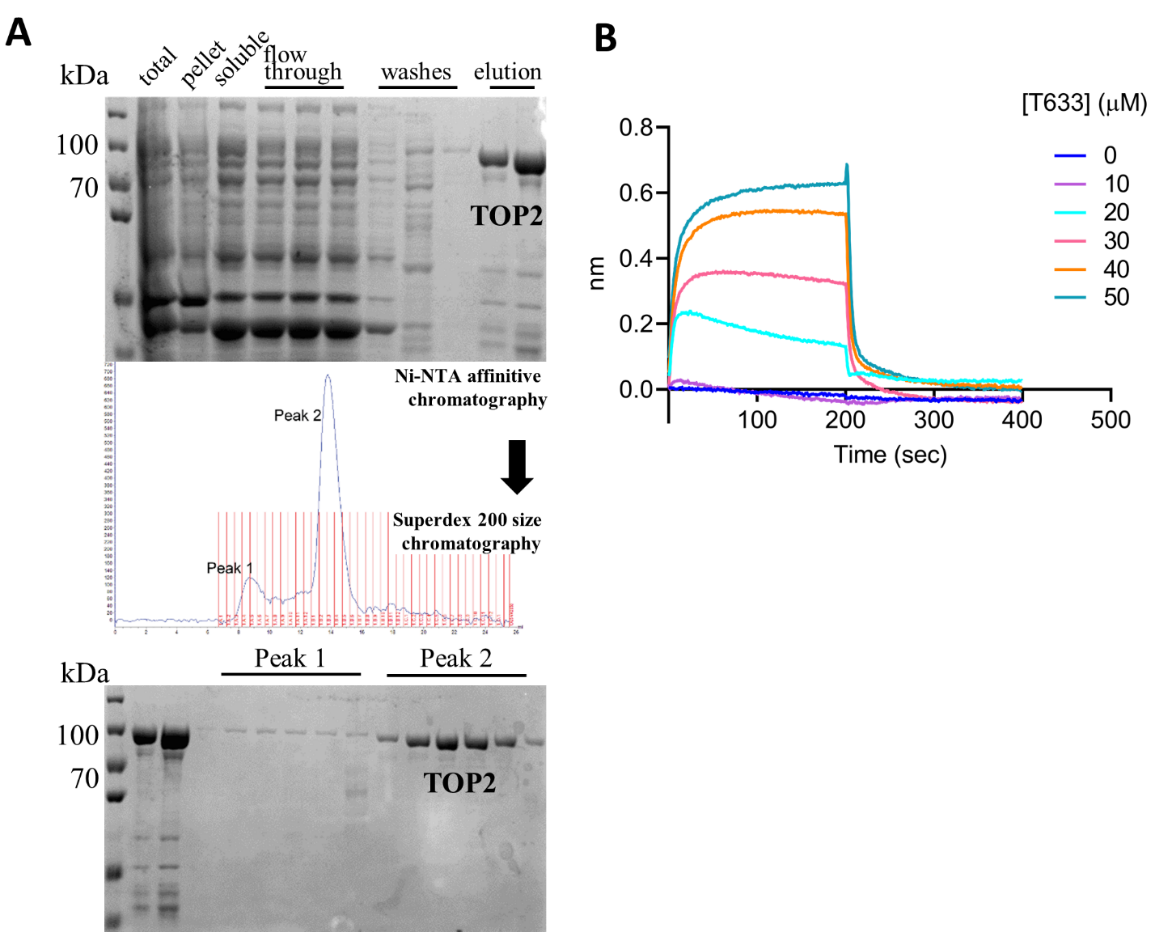
**

**Supplementary Figure 2. Purification of human TOP2A (431-1193) from bacteria to perform BLI assays. (A)** Human TOP2A (431-1193) was purified from Escherichia coli BL21 by metal ion affinity chromatography (IMAC) with nickel nitrilotriacetic acid (NiNTA) resin and cation-exchange chromatography (HiTrap SP). **(B)** BLI dose-response curves (0, 10, 20, 30, 40, and 50 μM) reflecting the direct and reversible binding of T633 with TOP2A (431-1193). The streptavidin biosensors (Fortbio) were pre-incubated with biotinylated TOP2A(431-1193), and then dipped in successive wells containing increasing concentrations of T633. The Kd was estimated to be 35 ± 3 µM with Hill slope of  5±1.5.


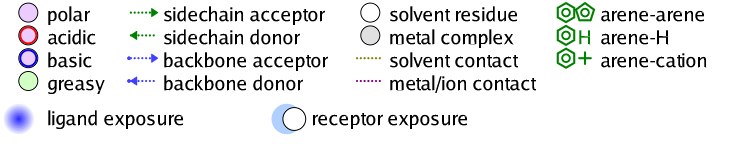

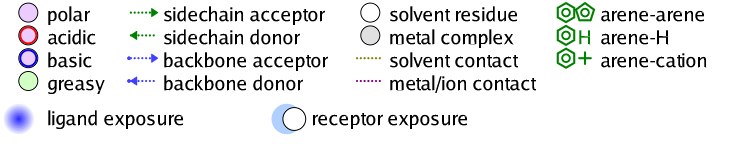


Numbers – bond energy (kcal/mol)


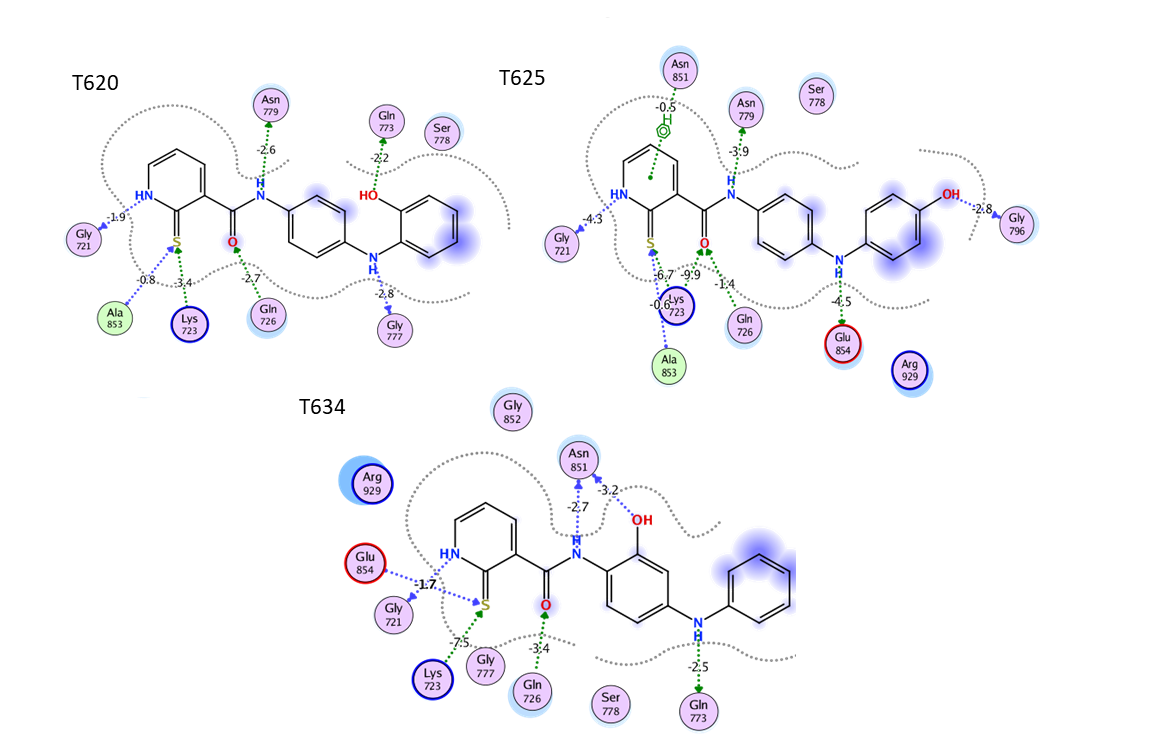


**Supplementary Figure 3. T60 derivatives interact with TOP2A.** Key protein-ligand interactions of the active compounds of T620, T625, and T634 with TOP2A.

**

**

**Supplementary Figure 4. T60 delays S and M phases of cell cycling.** HeLa cells were treated with vehicle or 20 µM of T60 for 48 hours. Cells were then stained with APC-BrdU and 7-AAD and subjected to FACS to measure cell populations at Sub G0/G1, G0/G1, S, and G2/M phases.

**

**

**Supplementary Figure 5. On-target effects of T60 demonstrated by chromatin fractionation assays.** K562 cells were treated with 0, 1, and 20 µM of T60 for 24 hours. Chromatin associated proteins and the whole-cell lysis were extracted and used to perform immunoblotting assays with TOP2A and TOP2B antibodies. Histone H3 and beta-actin were used as loading controls.

**

**

**Supplementary Figure 6. T60 inhibits the proliferation of multiple cancer cell models.** Various cancer cell lines were treated with increasing doses of paclitaxel, camptothecin, or etoposide alone or in combination with 0, 5, 10, 15, and 20 µM of T60. Cell viability was determined by MTS assays. Results were calibrated with vehicle treatment as 100%. The doses of paclitaxel, camptothecin, or etoposide were indicated in Supplementary Table 2.

**

**

**Supplementary Figure 7. T60 caused negligible DNA damages in cancer cells.** Cells were cultured in different conditions respectively. RPMI-1640 medium containing 10% FBS for U937, K562, Jurkat, THP, H69, H82, H446, and NCCIT. DMEM medium containing 10% FBS for DUNE, NT2, Hela, PC3, DU145. Cells were treated with DMSO, 5 µM of Paclitaxel, camptothecin, etoposide or T60 for 4 hours. The protein lysis was extracted and immunoblotted with γH2AX and β-Actin. Three independent experiments were performed, and one set of Western blotting images are presented.

**Table S1**

| **Cell Lines** | **PTX (nM)** | **CPT (nM)** | **T60 (µM)** |
| --- | --- | --- | --- |
| K562 | 5 | - | 5, 10, 15, 20 |
|  | 1, 2, 5,10 | - | 20 |
|  | - | 10 | 5, 10, 15, 20 |
|  | - | 5, 10, 20, 40 | 20 |
| U937 | 15 | - | 5, 10, 15, 20 |
|  | 5, 10, 15,20 | - | 20 |
|  | - | 100 | 5, 10, 15, 20 |
|  | - | 5, 40, 100, 200 | 20 |
| NCI-H82 | 4 | - | 5, 10, 15, 20 |
|  | 3, 4, 5, 10 | - | 20 |
|  | - | 6 | 5, 10, 15, 20 |
|  | - | 5, 10, 15, 10 | 20 |
| NCCIT | 3 | - | 5, 10, 15, 20 |
|  | 1, 2, 5,10 | - | 20 |
|  | - | 10 | 5, 10, 15, 20 |
|  | - | 5, 10, 20, 40 | 20 |

**Supplementary Table 1. Co-treatment of T60 with paclitaxel and camptothecin in cancer cell models.** K562, U937, NCI-H82, and NCCIT cancer cells were treated with paclitaxel or camptothecin alone or in combination with 0, 5, 10, 15, and 20 µM of T60. Cell viability was determined by MTS assays. Concentrations of each chemical in specific cell lines are indicated.

**Table S2**

| **Cell lines** | **Paclitaxel (nM)** | **CPT (nM)** | **T60 (µM)** |
| --- | --- | --- | --- |
| **Jurkat** | 20 | - | 5, 10, 15, 20 |
|  | 5, 10, 20, 30 | - | 20 |
|  | - | 10 | 5, 10, 15, 20 |
|  | - | 5, 10, 20, 40 | 20 |
| **THP** | 10 | - | 5, 10, 15, 20 |
|  | 1, 5, 10, 15 | - | 20 |
|  | - | 20 | 5, 10, 15, 20 |
|  | - | 5, 10, 20, 40 | 20 |
| **H69** | 15 | - | 5, 10, 15, 20 |
|  | 10, 15, 30, 50 | - | 20 |
|  | - | 80 | 5, 10, 15, 20 |
|  | - | 20, 40, 80, 100 | 20 |
| **H446** | 4 | - | 5, 10, 15, 20 |
|  | 3, 4, 5, 10 | - | 20 |
|  | - | 20 | 5, 10, 15, 20 |
|  | - | 5, 20, 50, 100 | 20 |
| **NT2** | 10 | - | 5, 10, 15, 20 |
|  | 5, 10, 20, 40 | - | 20 |
|  | - | 60 | 5, 10, 15, 20 |
|  | - | 20, 40, 60, 80 | 20 |
| **Hela** | 50 | - | 5, 10, 15, 20 |
|  | 10, 30, 50, 70 | - | 20 |
|  | - | 40 | 5, 10, 15, 20 |
|  | - | 20, 40, 60, 80 | 20 |

**Supplementary Table 2. Co-treatment of T60 with paclitaxel and camptothecin in cancer cell models.** Various cancer cells were treated with paclitaxel or camptothecin alone or in combination with 0, 5, 10, 15, and 20 µM of T60. Cell viability was determined by MTS assays. Concentrations of each chemical in specific cell lines are indicated.
